# Understory species composition mediates soil greenhouse gas fluxes by affecting bacterial community diversity in boreal forests

**DOI:** 10.3389/fmicb.2022.1090169

**Published:** 2023-01-20

**Authors:** Beixing Duan, Ruihan Xiao, Tijiu Cai, Xiuling Man, Zhaoxin Ge, Minglei Gao, Maurizio Mencuccini

**Affiliations:** ^1^School of Forestry, Northeast Forestry University, Harbin, China; ^2^Key Laboratory of Sustainable Forest Ecosystem Management-Ministry of Education, Northeast Forestry University, Harbin, China; ^3^CREAF, Barcelona, Spain; ^4^ICREA, Barcelona, Spain

**Keywords:** greenhouse gas, soil bacterial community, boreal forest, larch forest, understory species

## Abstract

**Introduction:**

Plant species composition in forest ecosystems can alter soil greenhouse gas (GHG) budgets by affecting soil properties and microbial communities. However, little attention has been paid to the forest types characterized by understory vegetation, especially in boreal forests where understory species contribute significantly to carbon and nitrogen cycling.

**Method:**

In the present study, soil GHG fluxes, soil properties and bacterial community, and soil environmental conditions were investigated among three types of larch forest [*Rhododendron simsii*-*Larix gmelinii* forest (RL), *Ledum palustre*-*Larix gmelinii* forest (LL), and *Sphagnum-Bryum*-*Ledum palustre*-*Larix gmelinii* forest (SLL)] in the typical boreal region of northeast China to explore whether the forest types characterized by different understory species can affect soil GHG fluxes.

**Results:**

The results showed that differences in understory species significantly affected soil GHG fluxes, properties, and bacterial composition among types of larch forest. Soil CO_2_ and N_2_O fluxes were significantly higher in LL (347.12 mg m^−2^ h^−1^ and 20.71 μg m^−2^ h^−1^) and RL (335.54 mg m^−2^ h^−1^ and 20.73 μg m^−2^ h^−1^) than that in SLL (295.58 mg m^−2^ h^−1^ and 17.65 μg m^−2^ h^−1^), while lower soil CH_4_ uptake (−21.07 μg m^−2^ h^−1^) were found in SLL than in RL (−35.21 μg m^−2^ h^−1^) and LL (−35.85 μg m^−2^ h^−1^). No significant differences between LL and RL were found in soil CO_2_, CH_4_, and N_2_O fluxes. Soil bacterial composition was mainly dominated by Proteobacteria, Actinobacteria, Acidobacteria, and Chloroflexi among the three types of larch forest, while their abundances differed significantly. Soil environmental variables, soil properties, bacterial composition, and their interactions significantly affected the variations in GHG fluxes with understory species. Specifically, structural equation modeling suggested that soil bacterial composition and temperature had direct close links with variations in soil GHG fluxes among types of larch forest. Moreover, soil NO_3_^−^−N and NH_4_^+^ − N content also affected soil CO_2_, CH_4_, and N_2_O fluxes indirectly, *via* their effects on soil bacterial composition.

**Discussion:**

Our study highlights the importance of understory species in regulating soil GHG fluxes in boreal forests, which furthers our understanding of the role of boreal forests in sustainable development and climate change mitigation.

## Introduction

Climate warming, resulting from the enrichment of the atmospheric greenhouse gases (GHG), has caused serious ecological and environmental issues at global scales. From the pre-industrial era, CO_2_, CH_4_, and N_2_O, as the major GHGs, account for 66, 16, and 7% of the increase in global radiative forcing, respectively (WMO, 2019). Soils hold the largest terrestrial carbon and nitrogen pools and 35% CO_2_, 47% CH_4_, and 53% N_2_O of the annual emissions are related to soil degassing (IPCC, 2007; Scharlemann et al., 2014). Thus, soil GHG emissions are key processes affecting the atmosphere GHG balance and global climate changes. Plant species composition has been identified to have large effects on soil GHG fluxes by changing plant characteristics, soil environmental factors, physicochemical properties, and microbial communities (Oertel et al., 2016; He et al., 2021) which can individually or interactively affect soil GHG emissions. In the past, global warming has caused variations in plant species composition and distribution by regulating plant growth and reproduction (Villén-Peréz et al., 2020; Yang et al., 2020) and increasing hazard frequency, such as fire, floods, or drought risk (Johnstone et al., 2010; Yigit et al., 2016; Moqimzai, 2020). Thus, variations in soil GHG fluxes and their associated drivers following plant species changes is a crucial research topic.

Forestlands cover about 28% of the area of terrestrial ecosystems and forest soils are generally important sources or sinks for CO_2_, N_2_O, and CH_4_ (Oertel et al., 2016). Thus, even minor variations in soil GHG fluxes may have obvious effects on climate change. Over past decades, a number of field studies have been conducted to clarify the effects of plant species changes on soil GHG fluxes in forest ecosystems (Chen et al., 2020; Hsieh et al., 2021; Lee et al., 2022). However, most studies focus on forest ecosystems dominated by different tree components (Liu et al., 2014; Mazza et al., 2021; Quebbeman et al., 2021). Little attention has been paid to components of the understory (e.g., dwarf shrubs, mosses, and lichens) on soil GHG fluxes. Actually, understory vegetation plays a vital role in regulating soil microclimate (Prévosto et al., 2020), properties (Yao et al., 2019), belowground processes (Nilsson and Wardle, 2005; Zhou et al., 2018), and soil microbial community structure (Xiao et al., 2022), which ultimately can affect the soil GHG fluxes in forest ecosystems, especially in boreal forest ecosystems. Specifically, boreal forests are characterized by a tree layer and an understory of short woody ericaceous shrubs, and frequently mosses and lichens (De Long et al., 2016). The understory vegetation here not only has higher relative productivity (comparable to that of the trees) but also has a high turnover rate which contributes substantially to soil nutrient cycling (Nilsson and Wardle, 2005; Xiao et al., 2020a). However, because of their negligible biomass (Xiao et al., 2020a), the potential ecological function of understory species on carbon and nitrogen cycling has usually been overlooked in boreal regions (Wu et al., 2019; Xiao et al., 2020b; Gao et al., 2021). This leads to significant uncertainty in the assessment of boreal forest response to climate change. Thus, it is crucial to explore how and to what extent soil GHG fluxes vary with understory vegetation and its potential control mechanisms in forest types with the same tree layer in boreal regions.

Some previous studies have attempted to explore the effects of understory species on soil GHG fluxes, but there are no consistent results. A study in chestnut plantations suggests that the replacement of natural understory vegetation with *Medicago sativa* L. and *Lolium perenne* L. will increase CO_2_ and N_2_O emissions and reduce CH_4_ uptake, while no significant differences in soil GHG fluxes are found between the two replacement understory species (Zhang et al., 2014). However, studies in four forest plantations show that the replacement of natural understory vegetation with *Cassia alata* has no significant effects on soil N_2_O and CH_4_ emissions (Li, 2010; Li et al., 2010). Previous studies in boreal forests show that the effects of understory vegetation on soil GHG fluxes are dependent on understory species (Li et al., 2018; Gao et al., 2022). These different results may be due to the differences in species traits. Generally, soil GHG emissions are controlled by biogeochemical processes driven by soil microbial communities (Oertel et al., 2016; Muhammad et al., 2022), which can be regulated by soil properties and micro-environmental conditions (Kolton et al., 2019; Zhu et al., 2020). Plants differ in litter and root traits, which are the main sources of soil organic matter and nutrients (De Long et al., 2016; Xiao et al., 2020a). Thus, differences in understory species can affect soil GHG fluxes *via* their effects on organic matter decomposition and nutrient cycling (De Long et al., 2016; Pan et al., 2018). Meanwhile, understory species can affect soil temperature and moisture by shading and its effect of evapotranspiration (Bond-Lamberty et al., 2011; Myers-Smith et al., 2011). Soil GHG fluxes are highly sensitive to soil temperature and moisture (Chen et al., 2021; Duan et al., 2022; Wang et al., 2022). However, the understory management experiments show that the effects of understory vegetation on soil temperature and moisture differ among understory species (Li et al., 2010; Zhang et al., 2015). Further, understory vegetation can also affect the soil microbial communities (Fu et al., 2015; Xiao et al., 2022), which drive the soil GHG consumption and regulation of biogeochemical processes (Oertel et al., 2016; Muhammad et al., 2022).

Soil CO_2_, CH_4_, and N_2_O fluxes also respond differently to edaphic factors (Chen et al., 2021). On the one hand, soil CO_2_ fluxes are mainly regulated by soil temperature; soil CH_4_ fluxes are more susceptible to soil moisture and an increase in soil moisture enhances anaerobic soil conditions, which favors soil CH_4_ production; while both soil temperature and moisture have significant impacts on soil N_2_O emissions (Song et al., 2021; Wang et al., 2022). On the other hand, soil nitrate-nitrogen (NO_3_^−^−N) and ammonium-nitrogen (NH_4_^+^ − N) are the main substrates of soil N_2_O production (Han and Zhu, 2020). However, higher NO_3_^−^−N and NH_4_^+^ − N can also inhibit soil heterotrophic respiration and CH_4_ oxidation, respectively (Kuzyakov and Xu, 2013; Liu Y. et al., 2017). Moreover, production and consumption mechanisms also differ among soil GHGs (Oertel et al., 2016). Soil respiration generally consists of autotrophic and heterotrophic respiration, and the latter is closely related to the effects of bacterial activity and abundance on soil organic matter degradation; soil CH_4_ fluxes are the balance of CH_4_ production by methanogens and consumption by methanotrophs; soil N_2_O is produced by both nitrification and denitrification, which are primarily driven by nitrifying and denitrifying bacteria, respectively (Hu et al., 2015; Oertel et al., 2016). These differences may induce soil CO_2_, CH_4_, and N_2_O fluxes to respond differently to understory variations. Thus, although soil GHG fluxes could vary with understory species in forest ecosystems, we still lack of a comprehensive understanding of how and to what extent changes in its primary drivers affect soil GHG fluxes.

In the present study, we conducted a field study to explore the variations in soil GHG fluxes with understory vegetation in different types of larch forest in boreal regions of China. Specifically, these forests have the same larch layer but different understory vegetations, including *Rhododendron simsii*-*Larix gmelinii* forest (RL), *Ledum palustre*-*Larix gmelinii* forest (LL), and *Sphagnum-Bryum*-*Ledum palustre*-*Larix gmelinii* forest (SLL), where the understory vegetations are dominated by *Rhododendron dauricum*, *Ledum palustre*, and *Sphagnum palustre* and *Bryum, respectively.* Soil GHG fluxes, properties, and bacterial community as well as soil environmental variables were measured to explore the potential processes controlling soil GHG fluxes variations. We hypothesized that (1) variations in understory species composition will have significant effects on soil properties and bacterial communities due to the specific plant traits, (2) these changes will lead to significant differences in soil CO_2_, CH_4_, and N_2_O fluxes among three types of larch forest, and (3) given the same larch tree cover, soil microbial-mediated processes associated with CO_2_, CH_4_, and N_2_O are mainly regulated by soil bacterial diversity rather than richness.

## Materials and methods

### Study area

The research took place in the Heilongjiang Mohe Forest Ecosystem Research Station (122°06′–122°27′E, 53°17′–53°30′N) of the Daxing’an Mountains, Northeast China. This area has a typical cold-temperate continental monsoon climate. The annual average temperature is −4.9°C and the annual average is 430–500 mm (Gao et al., 2019). The frost-free period spans approximately 80–90 days from June to August and snow cover is present for more than half a year (Duan et al., 2020). The maximum snow depth was 68.6 cm in the meteorological station (Lin et al., 2018). The primary soil type is brown coniferous forest soil, which is classified as Podzol according to the FAO soil classification.

This area belongs to the northern taiga, with cold temperate deciduous tree species larch (*Larix gmelinii* L.) as the dominant species. Meanwhile, the Mongolian pine (*Pinus sylvestris* var. *mongolica* L.) forest, birch (*Betula platyphylla* L.) forest, and aspen (*Populus davidiana* L.) forest are also distributed in this area. The understory species are mainly dominated by *Ledum palustre* L., *Rhododendron dauricum* L., *Vaccinium vitis*-*idaea* L., and *Sphagnum* sp. Here, due to different site conditions, the larch forests can be characterized by the same tree layer but different understory vegetations, such as the *Sphagnum-Bryum-Ledum palustre-Larix gmelinii* forest (SLL), *Rhododendron dauricum-Larix gmelinii* forest (RL), and *Ledum palustre-Larix gmelinii* forest (LL). Thus, three typical larch forests, including RL, LL, and SLL, were selected in this study. These three types of larch forest were naturally regenerated from burned land, with a slope < 5°. The basic characteristics of the three larch forests are shown in Table 1. In each larch forest, three 20 m × 30 m plots were placed randomly and a total of nine sample plots were established. In each plot, three 1 m × 1 m subplots (a total of nine independent subplots in each plot) free of trees were randomly selected for GHG and soil sampling.

**Table 1:** Stand characteristics of three types of larch forest.

Forest types	LL	RL	SLL
Stand age	83	83	83
Elevation (m)	326 ± 3	324 ± 3	332 ± 4
Stand density (tree ha^−1^)	1,300 ± 100	1,270 ± 130	1,120 ± 130
Soil organic carbon stock (Mg ha^−1^)	52.54 ± 7.07b	38.54 ± 5.93c	94.99 ± 11.87a
Soil total nitrogen stock (Mg ha^−1^)	3.06 ± 0.43b	2.19 ± 0.28c	7.04 ± 0.78a
Soil pH	5.50 ± 0.05c	5.73 ± 0.05a	5.61 ± 0.02b
Soil bulk density (g cm^−3^)	0.86 ± 0.07b	0.72 ± 0.03c	1.34 ± 0.11a

The soil characteristics were measured in 0–10 cm soil depth. The different lowercase letters behind the values meant significant difference among three types of larch forest.

### Soil GHG fluxes

In the present study, soil GHG fluxes were measured by the static opaque chamber technique. In September 2018, a total of 27 permanent open square-framed base collars (50 cm × 50 cm) with a groove in the upper edge made of polypropylene were inserted into the mineral soil to approximately 5 cm depth. The base collars were set exactly in the middle in each subplot, respectively, with one collar in each subplot. During the study period, the aboveground biomass of shrubs and grasses in each subplot were removed by clipping when setting the base collars to minimize autotrophic respiration. The experiment was conducted during the snow-free period from May to October in 2019. During sampling, an opaque chamber (50 cm × 50 cm × 50 cm) was carefully placed on the base collar to avoid inducing air pressure pumping. Insulation material was fixed outside of the opaque chamber to avoid temperature changes during flux measurements. GHG fluxes were measured twice per month, but we only measured once in June, August, and October due to the weather conditions (rainfall and snowfall). Thus, nine events were recorded in total. Sampling took place between 9:00 and 11:00 a.m., when the soil GHG fluxes are recognized to best represent the daily mean (Alves et al., 2012; Gao et al., 2022).

Gas samples were collected with a 50-ml plastic syringe equipped with a three-way stopcock through the rubber septum in the lid at 0, 15, 30, and 45 min (a total of four samples during each event) after chamber closure. Gas samples were injected into a 100 ml pre-evacuated gas sampling bag (Delin Gas Packing Co., Dalian, China) and transported to the lab for gas analysis. Simultaneously, we measured the air temperature inside the chamber when the gas sample was collected. Gas samples were stored at air temperature and analyzed within 1 week. Gas concentrations in all samples were determined with a gas chromatograph (TRACE 1300 GC, Thermo Fisher Scientific, United States). Fluxes were determined based on linear regression analysis of the change in gas concentration in the chambers with time over a 45-min period for each chamber. The fluxes for further analysis were selected with strong linear relationships (*r*^2^ > 0.75; Doroski et al., 2019).

Greenhouse gas flux rates for each chamber were calculated using the following formula:


(1) $$F=\frac{\mathrm{dc}}{\mathrm{dt}}\times \frac{M}{V_{0}}\times \frac{P}{P_{0}}\times \frac{T_{0}}{T}\times H$$

Where F is the flux of the respective greenhouse gas (mg m^−2^ h^−1^ for CO_2_, μg m^−2^ h^−1^ for CH_4_ and N_2_O), dc/dt is the slope of the linear regression for the gas concentration gradient over time (μmol mol^−1^ h^−1^); M is the molecular mass of gas (g mol^−1^); V_0_ is the gas molar volume (m^3^ mol^−1^); P and P_0_ are the atmospheric pressure (Pa) and atmospheric pressure under standard conditions (Pa), respectively; T and T_0_ are the mean value of the air temperature inside the chamber during sampling (K) and absolute air temperature (K), respectively; and H is the effective height of chamber (m). Average gas flux and standard deviation were calculated from three replicates for each plot. Positive fluxes represent net soil GHG emissions and negative fluxes represent net soil GHG uptake.

### Global warming potential

Greenhouse gas global warming potential (GWP) can be used to assess and compare the potential climate impact of the emissions of different GHGs (IPCC, 2007). The comprehensive GWP of soil CO_2_, CH_4_, and N_2_O in each larch forest was calculated as follow:


(2) $$\mathrm{GWP}=C_{{\mathrm{CO}}_{2}}+27.2\times C_{{\mathrm{CH}}_{4}}+273\times C_{N_{2}O}$$

Where the $C_{{\mathrm{CO}}_{2}}$ , $C_{{\mathrm{CH}}_{4}}$ , and $C_{N_{2}O}$ were the cumulative fluxes of soil CO_2_, CH_4_, and N_2_O, respectively. When the GWP was estimated, the CO_2_ is recognized as the reference gas. Thus, the CH_4_ and N_2_O are converted into “CO_2_-equivalents.” Based on 100-year time horizon, the GWP of CH_4_ and N_2_O are 27.2 and 273 times of CO_2_ (IPCC, 2021).

### Soil samples and analysis

Soil samples were collected from the three types of larch forest at all 27 subplots when collecting the gas sample each time. For each sample subplot, one soil sample was collected at the depth of 0–10 cm using a stainless-steel corer in the vicinity of each chamber. Before soil cores were taken, the litter layer was carefully moved aside and only mineral soil was sampled. Soil samples from the same plot were mixed to form a composite sample. Thus, there were three composite samples per larch forest at each sampling time. The composite samples were stored in plastic bags and all stones, roots, and debris were removed carefully by hand. Then, all the samples were immediately transported in an insulated box to the laboratory for further study. Meanwhile, we also measured the soil temperature at 10 cm soil depth (ST) using a portable thermometer at each subplot (Delta TRAK, CA, United States).

Then the soil samples were separated into two parts after passing through a 2-mm screen. One was stored at 4°C for soil microbial biomass and soil inorganic nitrogen analysis; another part was air-dried at room temperature (20°C) for other analyses of soil properties. In this study, soil water content (SWC) was determined gravimetrically by oven drying the whole soil at 105°C for 24 h. Soil pH was determined using a pH meter equipped with a calibrated combined-glass electrode with the soil-to-water mass ratio of 1:2.5. Soil nitrate-nitrogen (NO_3_^−^−N) and ammonium-nitrogen (NH_4_^+^ − N) were measured by extraction with 1 mol L^−1^ KCl and the extraction suspensions analyzed using a flow injection auto-analyzer (Seal Analytical AA3, Norderstedt, Germany). Soil microbial biomass carbon (MBC) and nitrogen (MBN) were measured by the fumigation extraction method (Vance et al., 1987). Meanwhile, in the middle of July, three soil samples were collected in each plot individually. Soil samples at 0–10 cm depth from the same plot were mixed and stored in sterile centrifuge tubes. Then these soil samples were transferred immediately to the laboratory in an insulated box (Esky) on dry ice for DNA extraction.

### Soil DNA extraction and quantitative PCR analysis

Microbial DNA from samples was extracted using a PowerSoil® DNA Isolation Kit (MoBio Inc., Carlsbad, United States). DNA concentration and purity were monitored on 1% agarose gel. According to the concentration, DNA was diluted to 1 ng/μl using sterile water. The V3–V4 region of bacterial 16S rRNA genes was amplified using the specific primer (primer: 338F/806R: 5′-ACTCCTACGGGAGGCAGCAG-3′/5′-GGACTACHVGGGTWTCTAAT-3′) with the barcode. All PCR reactions were carried out in 30 μl reactions with 15 μl of Phusion®High-Fidelity PCR Master Mix (New England Biolabs); 0.2 μM of forward and reverse primers, and about 10 ng template DNA. Thermal cycling consisted of initial denaturation at 95°C for 5 min, followed by 27 cycles of denaturation at 94°C for 30 s, and at 72°C for 45 s. Finally 72°C for 10 min. The PCR products were purified with GeneJET Gel Extraction Kit (Thermo Fisher Scientific, United States) sequenced on the Illumina Hiseq 2500 platform (Magigene Co., Ltd., Guangzhou, China). Sequence analysis was performed using the QIIME 1.6.0 pipeline software. Sequences were assigned to operational taxonomic units (OTUs) with 97% similarity using Uparse software. The original sequence retrieved in this study has been deposited with the National Center for Biotechnology Information (NCBI) under the accession number PRJNA797560.

### Statistical analysis

One-way ANOVA followed by the LSD test was performed to test the differences in soil microenvironmental variables, properties, and soil GHG fluxes among three types of larch forest at *p* < 0.05 level. Two-way ANOVA was used to examine the effects of forest type and seasons, and their interactions on soil GHG fluxes and soil variables. Principal coordinates analysis (PCoA) based on Bray–Curtis was used to visualize soil bacterial community similarity among different types of larch forest. Pearson correlation analysis was used to explore the relationship between soil GHG fluxes and measured factors. Variation partitioning analysis (VPA) was used to identify the contribution of soil properties (soil NH_4_^+^-N, NO_3_^−^-N, MBC, and MBN), soil microenvironmental variables (soil temperature and soil water content), and soil bacterial community to soil GHG fluxes. The VPA was conducted in R software (Version 4.0.3) using the *vegan* package. Based on the VPA analysis, structural equation modeling (SEM) was used to explore whether the major pathways of soil factors and bacterial community affected soil GHG fluxes among three types of larch forest. The best fit model was assessed by fit indices, including non-significant paths (*p* > 0.05) and χ^2^ test (0 ≤ χ^2^/df ≤ 2), and standardized root mean square residual (SRMR < 0.05). The bacterial community was represented using the scores of the first dimensions in the PCA plot in SEM. The SEM analysis was conducted using AMOS 20.0 software (SPSS Inc., Chicago, IL, United States). The statistical analyses were performed in SPSS (19.0 for Windows, SPSS Institute, Inc., Chicago, IL, United States) and R software (Version 4.0.3). The figures were drawn with OriginPro 2016 (OriginLab Corp., Northampton, MA, United States).

## Results

### Soil physicochemical properties among three types of larch forest

**Figure 1:**
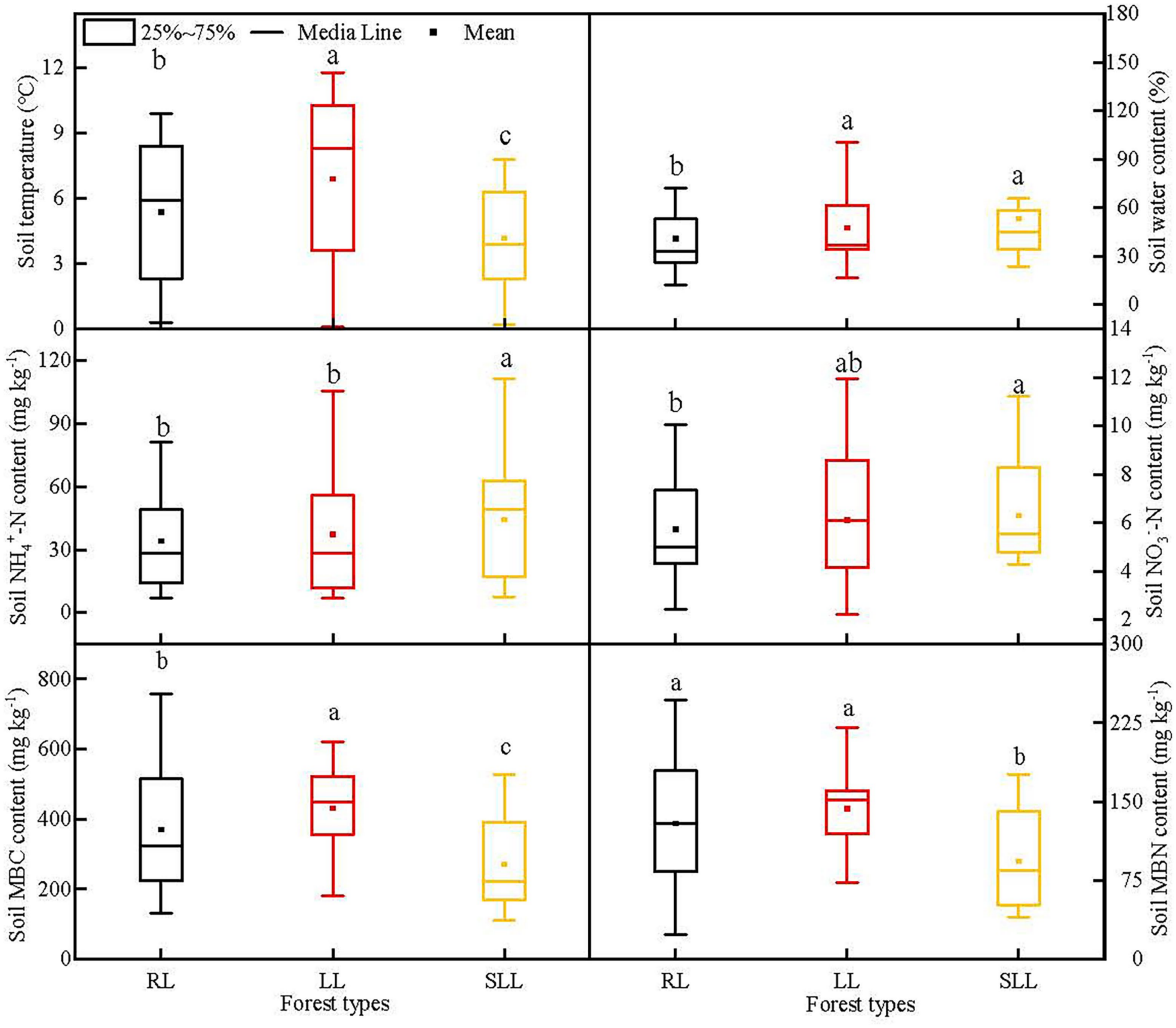
Soil environmental factors, soil inorganic nitrogen, and microbial biomass contents among three types of larch forest. LL, *Ledum palustre-Larix gmelinii* forest; RL, *Rhododendron dauricum-Larix gmelinii* forest; and SLL, *Sphagnum-Bryum-Ledum palustre-Larix gmelinii* forest. Different lowercase letters indicate statistically significant differences among different types of larch forest.

Mean soil temperature was highest in LL (6.89°C), then followed by RL (5.36°C), and lowest in SLL (4.17°C; *p* < 0.01). The highest mean soil water content was found in SLL (53.18%) and LL (47.35%) which were significantly higher than in RL (40.71%; *p* < 0.01). However, no significant differences in soil water content were found between LL and SLL (*p* > 0.05). Significant differences in soil properties were found among the three types of larch forest (Figure 1; *p* < 0.05). The soil NH_4_^+^-N content was highest in SLL (44.29 mg kg^−1^), significantly higher than in LL (37.27 mg kg^−1^) and RL (34.07 mg kg^−1^; *p* < 0.05), whereas there were no significant differences between the last two forest types (*p* > 0.05). Soil NO_3_^−^-N content in SLL was significantly higher than that in RL (*p* < 0.05), while no significant difference was found between LL and SLL or RL (*p* > 0.05). Both soil MBC and MBN contents ranked in the order of LL > RL > SLL (*p* < 0.01), whereas no significant difference in soil MBN content was found between LL and RL (*p* > 0.05).

### Soil GHG fluxes among three types of larch forest

**Figure 2:**
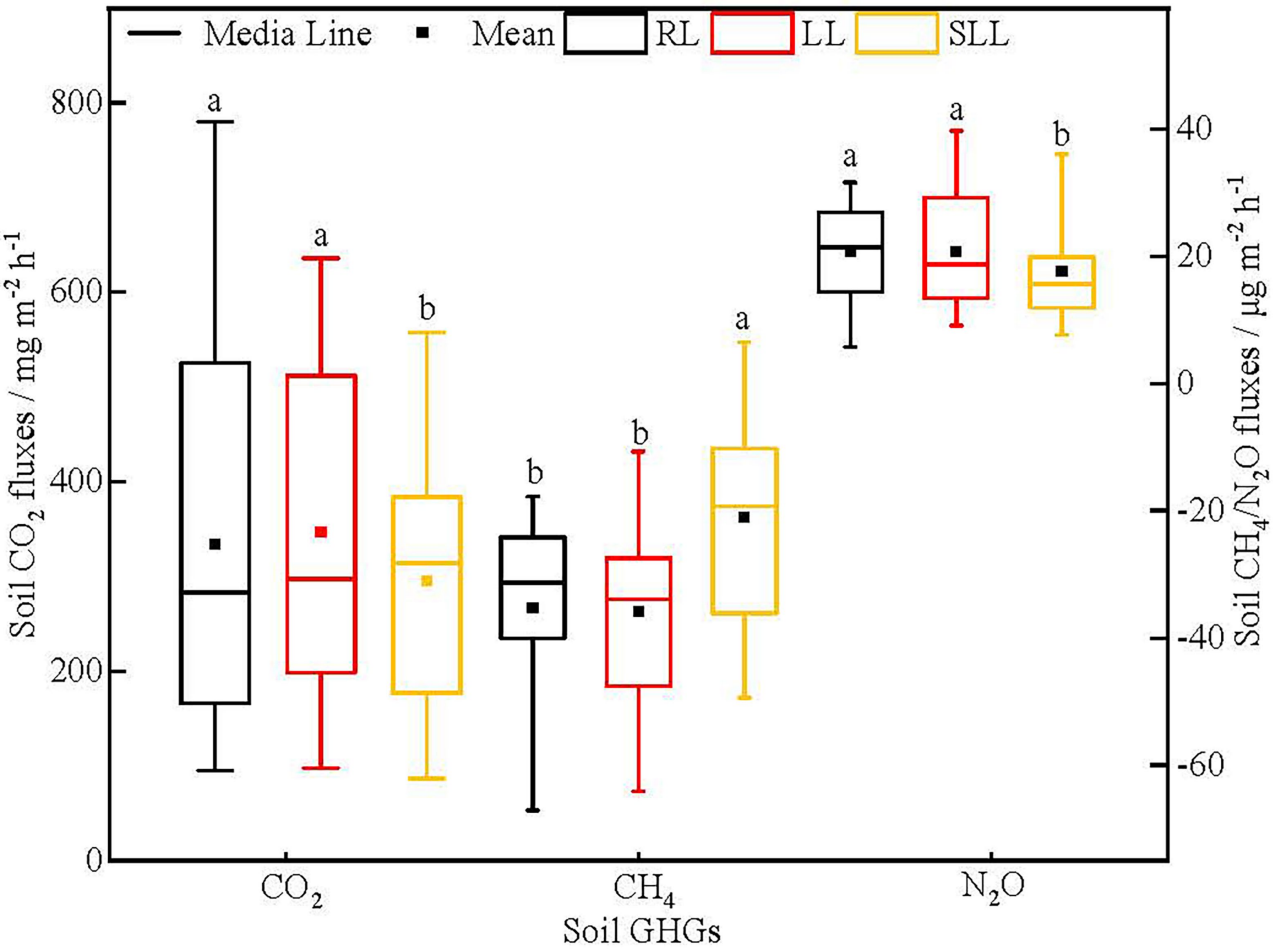
Variations in soil GHG fluxes among three types of larch forest. The different lowercase letters (a, b) indicate significant differences among three types of larch forest. Abbreviations: LL, *Ledum palustre-Larix gmelinii* forest; RL, *Rhododendron dauricum-Larix gmelinii* forest; and SLL, *Sphagnum-Bryum-Ledum palustre-Larix gmelinii* forest.

Soil GHG fluxes were significantly different among three types of larch forest (Figure 2). The soil CO_2_ fluxes in LL (347.12 mg m^−2^ h^−1^) and RL (333.54 mg m^−2^ h^−1^) were significantly higher than that in SLL (295.58 mg m^−2^ h^−1^; *p* < 0.05). However, no significant differences in soil CO_2_ fluxes were found between LL and RL (*p* > 0.05). The CH_4_ flux in SLL was-21.07 μg m^−2^ h^−1^ which was significantly higher than in LL (−35.85 μg m^−2^ h^−1^) and RL (−35.21 μg m^−2^ h^−1^; *p* < 0.01) and no significant difference was found between the last two types of larch forest (*p* > 0.05). Similarly, the soil N_2_O fluxes in LL and RL were 20.71 and 20.73 μg m^−2^ h^−1^, respectively, significantly higher than that in SLL (17.65 μg m^−2^ h^−1^; *p* < 0.05), whereas there were no significant differences between RL and LL (*p* > 0.05).

### Global warming potential

During the study period, there were significant differences in the cumulative soil GHG fluxes and their GWP among the three types of larch forest, which were consistent with the differences in average soil GHG flux (Table 2; Figure 2). The comprehensive GWP of soil GHG fluxes in LL (13.59 Mg CO_2_-Eq ha^−1^) and RL (12.89 Mg CO_2_-Eq ha^−1^) were significantly higher than in SLL (11.42 Mg CO_2_-Eq ha^−1^; *p* < 0.05), while no significant difference was found between LL and RL (*p* > 0.05). The contribution of CO_2_, CH_4_, and N_2_O to GWP was significantly different. The comprehensive GWP among three types of larch forest was mainly determined by the cumulative soil CO_2_ fluxes. The GWP of cumulative soil CH_4_ was negative among the three types of larch forest.

**Table 2:** Cumulative soil GHG fluxes and their GWP among three types of larch forest.

Forest types	Cumulative soil GHG fluxes			GWP (Mg CO_2_ Eq·ha^−1^)			
	CO_2_ (Mg ha^−1^)	CH_4_ (kg ha^−1^)	N_2_O (kg ha^−1^)	CO_2_	CH_4_	N_2_O	Comprehensive
LL	13.14 ± 1.18a	−1.39 ± 0.09b	0.80 ± 0.03a	13.14 ± 1.18a	−0.04 ± 0.00b	0.22 ± 0.01a	13.59 ± 1.19a
RL	12.89 ± 0.53a	−1.36 ± 0.11b	0.80 ± 0.05a	12.89 ± 0.53a	−0.04 ± 0.00b	0.22 ± 0.01a	13.06 ± 0.52a
SLL	11.42 ± 0.36b	−0.81 ± 0.08a	0.68 ± 0.05b	11.42 ± 0.36b	−0.02 ± 0.00a	0.19 ± 0.01b	11.59 ± 0.34b

Values are the mean ± SD. Different lowercase letters behind the number indicate statistically significant difference among different types of larch forest. LL, *Ledum palustre*-*Larix gmelinii* forest; RL, *Rhododendron dauricum*-*Larix gmelinii* forest; and SLL, *Sphagnum-Bryum-Ledum palustre-Larix gmelinii* forest.

### Characteristics of soil bacterial community among three types of larch forest

**Figure 3:**
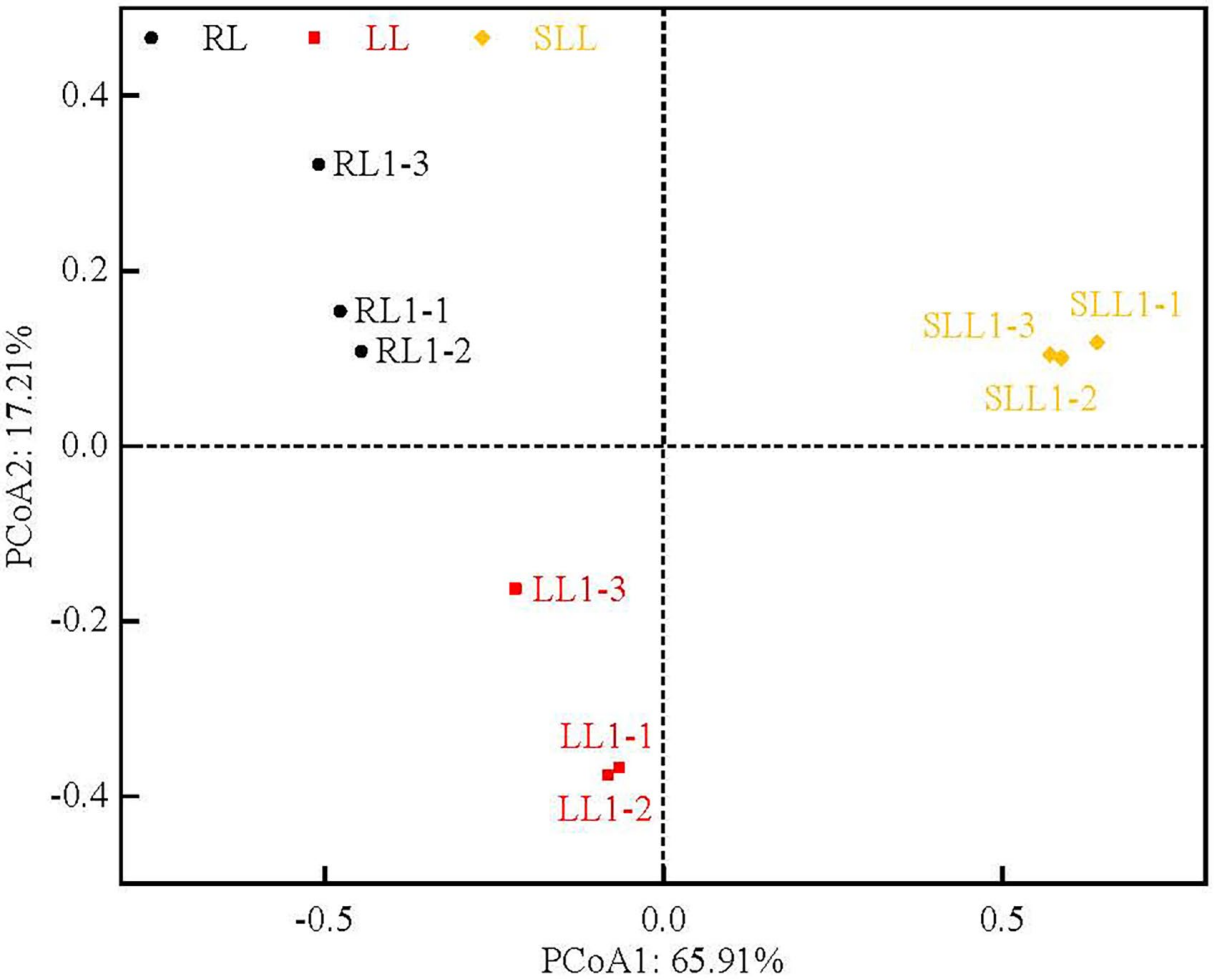
Principal component analysis (PCoA) of soil bacterial community diversity based on weighted UniFrac distances among three types of larch forest. LL, *Ledum palustre-Larix gmelinii* forest; RL, *Rhododendron dauricum-Larix gmelinii* forest; and SLL, *Sphagnum-Bryum-Ledum palustre-Larix gmelinii* forest.

Soil bacterial community composition was clearly clustered based on the types of larch forest (Figure 3). Differences in understory species composition caused some changes in bacterial community diversity. PC1 and PC2, the first two principal components, explained 65.91 and 17.21% of the total bacterial variability, respectively. The alpha diversity indices described the bacterial community richness (the Chao 1 index, based on abundance) and diversity (the Shannon and Simpson indices; Table 3). The Chao 1 index showed no significant differences among three types of larch forest (*p* > 0.05). However, there was a significantly higher Shannon index in LL than that in RL and SLL (*p* < 0.05). The Simpson indices in LL and SLL were lower than in RL (*p* < 0.05).

**Table 3:** OTU number, estimated indices of richness (Chao 1) and diversity (Shannon, Simpson, and Chao 1) and coverage among three types of larch forest.

Forest types	OTUs	Richness	Diversity		Coverage
		Chao1	Shannon	Simpson	
LL	2628.67 ± 24.96a	3159.67 ± 158.12a	6.73 ± 0.08a	0.0031 ± 0.001b	0.98 ± 0.01a
RL	2591.67 ± 50.08a	3004.33 ± 154.79a	6.45 ± 0.14b	0.0067 ± 0.002a	0.99 ± 0.00a
SLL	2743.00 ± 152.66a	3284.67 ± 115.38a	6.48 ± 0.04b	0.0054 ± 0.000ab	0.99 ± 0.00a

Values are the mean ± SD. Different lowercase letters behind the number indicate statistically significant difference among different types of larch forest. LL, *Ledum palustre*-*Larix gmelinii* forest; RL, *Rhododendron dauricum*-*Larix gmelinii* forest; SLL, *Sphagnum-Bryum-Ledum palustre-Larix gmelinii* forest.

**Figure 4:**
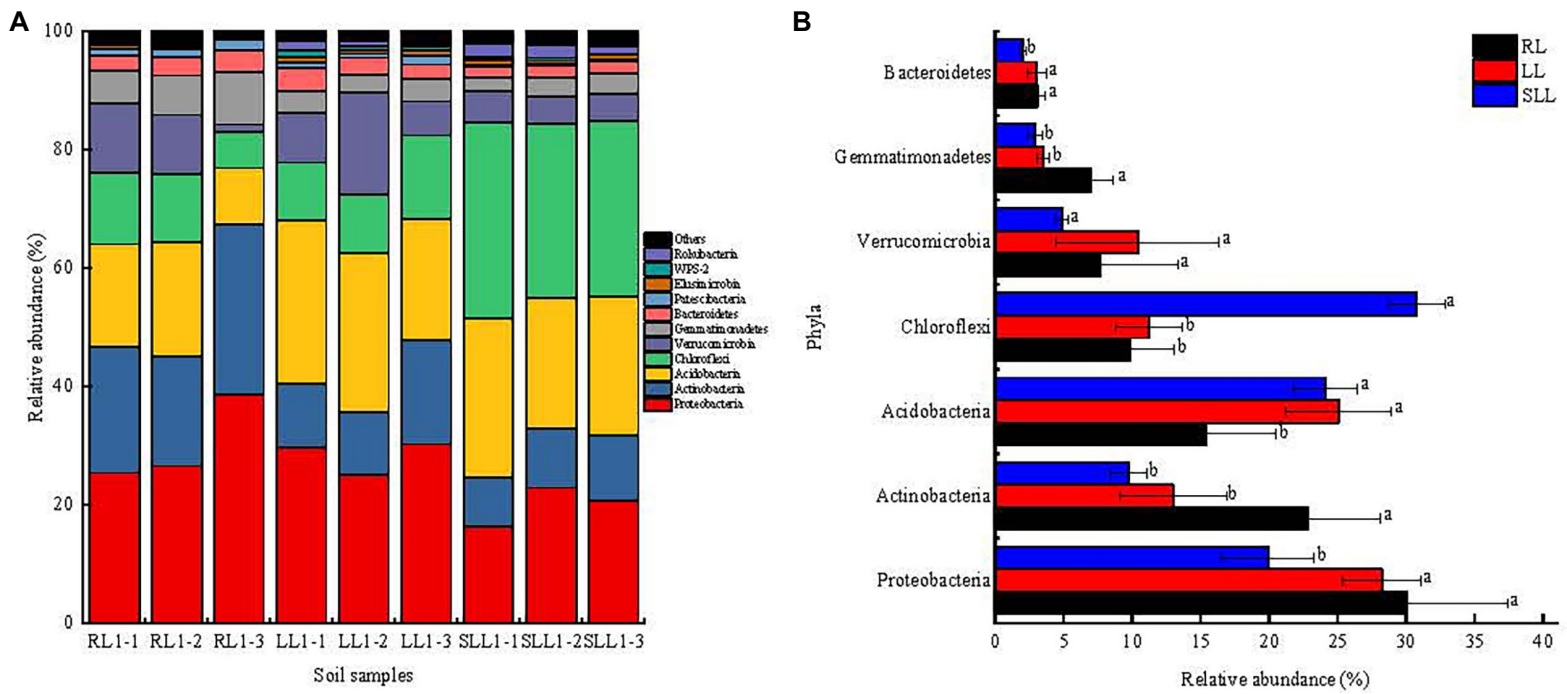
The relative abundance of soil bacterial taxonomic groups at phyla levels **(A)** and differences in the relative abundances of major bacterial community at the phyla **(B)**. Values are the mean ± SD. Different lowercase letters indicate statistically significant differences among different types of larch forest. LL, *Ledum palustre-Larix gmelinii* forest; RL, *Rhododendron dauricum-Larix gmelinii* forest; and SLL, *Sphagnum-Bryum-Ledum palustre-Larix gmelinii* forest.

Further, there were similar bacterial community structures at phylum level, but their relative abundance was different among three types of larch forest (Figure 4A). The dominant bacterial phyla were Proteobacteria, Actinobacteria, Acidobacteria, and Chloroflexi among all types of larch forest (Figure 4A). However, there were significant differences in their abundance (Figure 4B). The relative abundance of Proteobacteria in RL and LL reached 25.35–38.48 and 24.97–30.17%, significantly higher than that in SLL (16.25–22.81%; *p* < 0.05). Meanwhile, the relative abundance of Actinobacteria in RL (18.51–28.73%) was significantly higher than that in LL and SLL (*p* < 0.05) whereas no significant difference was found between the late two forest types (*p* > 0.05). However, the relative abundance of Acidobacteria in SLL (22.13–26.72%) and LL (20.63–27.67%) was significant higher than in RL (*p* < 0.05). Moreover, the highest Chloroflexi abundance was found in SLL (29.34–33.15%), but no significant differences were found between LL and RL (*p* > 0.05). There were no significant differences in Verrucomicrobia abundance among three types of larch forest (*p* > 0.05). However, the highest Gemmatimonadetes abundance was found in RL, and the lowest Bacteroidetes abundance in SLL (Figure 4B; *p* < 0.01).

### Drivers over soil GHG fluxes among three types of larch forest

**Figure 5:**
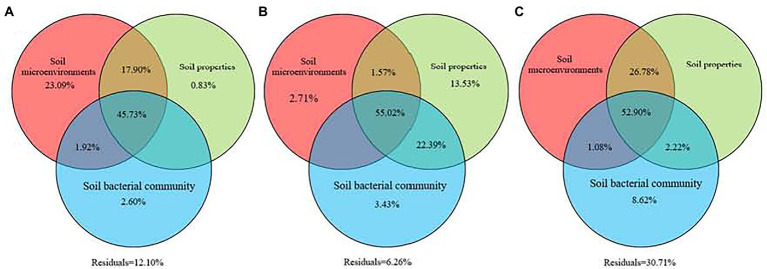
Relative contributions of soil microenvironments, soil properties, and soil bacterial community to greenhouse gas fluxes. Variation partitioning analysis was conducted to identify the variance in the CO_2_ **(A)**, CH_4_ **(B)**, and N_2_O **(C)** emissions explained by these three groups of biotic and abiotic factors. Values <0 are not shown.

Variations in soil GHG fluxes over the three types of larch forest were driven by soil environmental factors, soil properties, soil bacterial community, and their interactions (Figure 5). Variation partitioning analysis showed the interactions of soil environmental factors, soil properties, and soil bacterial composition contributed 47.18, 66.50, and 52.51% of variations in soil CO_2_, CH_4_, and N_2_O fluxes among three types of larch forest (Figure 5). Meanwhile, the relative importance of soil factors differed in regulating different soil GHG fluxes. Soil environmental factors, soil properties, and soil bacterial community played important roles in regulating soil CO_2_, CH_4_, and N_2_O fluxes alone, respectively.

**Figure 6:**
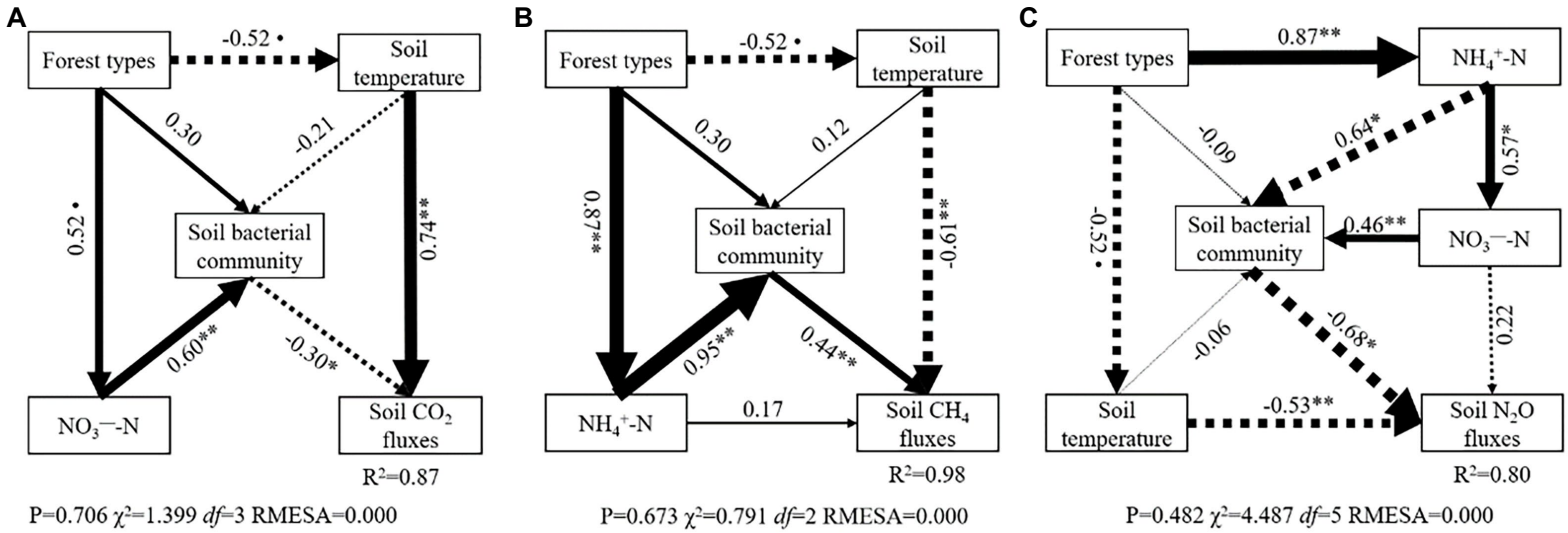
Structural equation model (SEM) depicting the effect of forest types on soil CO_2_ **(A)**, CH_4_ **(B)**, and N_2_O **(C)** fluxes. Single arrows represent direct linear causal relationships. Numbers beside arrows are standardized path coefficients. Arrow width is proportional to the size of the standardized path coefficients. Solid and dotted arrows indicate positive and negative relationships, respectively. The arrow width is proportional to the strength of the relationship. R2 denotes the proportion of variance explained by SEM. * and ** represented the significant correlation at *p* < 0.05 and *p* < 0.01 level.

Structural equation models (SEM) further indicated the key pathways through which understory changes regulated soil GHG flux variations among three types of larch forest (Figure 6). SEM explained 87, 98, and 80% of the variation in soil CO_2_, CH_4_, and N_2_O fluxes, respectively. Soil bacterial community and soil temperature were the main drivers in regulating directly CO_2_, CH_4_, and N_2_O flux variations among three types of larch forest. Meanwhile, soil inorganic nitrogen content (NH_4_^+^-N and NO_3_^−^-N) played a vital role in regulating soil GHG fluxes. Specifically, soil NO_3_^−^-N content affect soil CO_2_ fluxes indirectly by regulating soil bacterial community (Figure 6A). Soil NH_4_^+^-N also affected soil CH_4_ fluxes *via* its effect on soil bacterial community (Figure 6B). Both soil NH_4_^+^-N and NO_3_^−^-N content had significant indirect effects on soil N_2_O fluxes variations *via* their effects on soil bacterial community (Figure 6C).

## Discussion

In boreal forests, feather mosses and ericaceous dwarf shrubs are believed to have significant effects on soil microclimate and nutrients by shading and altering litter quality and decomposition rates (Myers-Smith et al., 2011; De Long et al., 2016). Our study further supports their finding, implying that soil microenvironmental factors and properties significantly differed among three types of larch forest. Meanwhile, soil bacterial composition also showed significant differences. However, the alpha diversity index showed that differences in understory species only altered soil bacterial diversity but had no significant effect on bacterial richness among three types of larch forest (Table 3). This phenomenon is likely because soil bacterial community is mainly shaped by the dominant plant species (Wang et al., 2018), suggesting that the larch layer defined the soil bacterial community richness in our study. The VPA results showed soil microenvironmental factors, properties, bacterial community, and their interactions contributed to variations in soil GHG fluxes among three types of larch forest (Figure 5), which agree with the first hypothesis in our study. However, variations in soil CO_2_, CH_4_, and N_2_O were primarily regulated by different factors (Figure 6).

### Effect of understory species on soil CO_2_ fluxes

As we expected, differences in understory species significantly altered soil CO_2_ fluxes among three types of larch forest. This point is consistent with previous studies (Zhang et al., 2014; Liu et al., 2019), suggesting that changes in understory shrubs biomass correlate to the spatial variation of soil respiration and understory replacement enhances soil GHG emission. Variations in soil CO_2_ fluxes with understory species were directly linked to soil bacterial composition and soil temperature (Figure 6A). Proteobacteria, Bacteroidetes, Acidobacteria, Chloroflexi, and Actinobacteria at the phylum level are believed to play major roles in the degradation of soil organic matter and carbon cycling (Xiao et al., 2017; Jia et al., 2018; Zhang et al., 2022). Proteobacteria and Bacteroidetes are believed to have positive correlations with soil organic matter mineralization (Fierer et al., 2007). Thus, the significantly higher abundance of Proteobacteria and Bacteroidetes led to higher soil CO_2_ emission in LL and RL than that in SLL in our study (Figure 4B). Acidobacteria and Actinobacteria have ability to degrade recalcitrant organic carbon (Trivedi et al., 2013). Although there were significant differences in both Acidobacteria and Actinobacteria abundance (Figure 4B), no significant difference was found in total Acidobacteria + Actinobacteria abundance among three types of larch forest. This means that differences in soil CO_2_ fluxes among the three types of larch forest may not come from the degradation of recalcitrant organic matter. Meanwhile, Chloroflexi has been intensified to perform a unique C fixation metabolism (Shih et al., 2017; He et al., 2021). Thus, the higher Chloroflexi abundance in SLL indicated the higher C fixation rather than CO_2_ emission in SLL than that in LL and RL (Figure 4B), which is another reason resulting in the lower soil CO_2_ emission in SLL. Partly agreeing with our hypothesis two, there were no significant differences in soil CO_2_ fluxes between RL and LL, which can also be explained by their similar bacterial abundance. Apart from soil bacterial communities, soil fungal communities also have potential effects on CO_2_ fluxes (Lee et al., 2022). We could not determine fungal community composition in our study, but our measurements of microbial biomass C and N include also this biota. The higher MBC content did not cause higher soil CO_2_ fluxes, which may be due to the differences in soil fungal communities (Liu et al., 2021). Meanwhile, compared to bacteria, soil fungal communities are sensitive to litter composition (Habtewold et al., 2020). Thus, the analysis of fungal communities among three types of larch forest remains an important area for future research.

Moreover, soil temperature and NO_3_^−^−N content also had significant effects on soil CO_2_ fluxes directly and indirectly, respectively. High soil temperature can accelerate the turnover rates of soil bacterial communities (Wu et al., 2015). Thus, higher soil temperature in LL and RL can lead to higher soil CO_2_ fluxes than SLL. There was higher soil temperature in LL than in RL, but the higher soil water content in LL may offset the effects of soil temperature on soil CO_2_ emission and ultimately induce similar soil CO_2_ fluxes between them (Wu et al., 2019). It has been well suggested that soil NO_3_^−^−N content is closely related to soil respiration (Chen et al., 2021) because NO_3_^−^−N can inhibit the activity of the enzymes in the soil (Li et al., 2015). Meanwhile, boreal forests are nitrogen limited ecosystems, and the availability of this nutrient is essential to organic matter decomposition and to soil bacterial metabolism (Nordborg et al., 2007; Sponseller et al., 2016). Thus, the higher soil NO_3_^−^−N content is another potential reason explaining the lower soil CO_2_ fluxes in SLL than that in RL.

### Effect of understory species on soil CH_4_ fluxes

The forest soils exhibited net CH_4_ uptake with the mean CH_4_ fluxes ranging from −35.85 to −21.07 μg m^−2^ h^−1^ among three types of larch forest. This result is consistent with previous studies which suggest the larch forest soils show a net sink of atmospheric CH_4_ in this study area (Wu et al., 2019; Duan et al., 2022). However, the capacity of soil net CH_4_ uptake showed significant differences in RL, LL, and SLL. Particularly, the soil CH_4_ fluxes in SLL were significantly higher than that in RL and LL, whereas no significant differences were found between LL and RL (Figure 2), which partly agrees with our hypothesis 2. In the present study, differences in understory species affected soil CH_4_ fluxes by regulating directly soil bacterial community and soil temperature among three types of larch forest (Figure 6B). Soil CH_4_ fluxes are the balance of production and consumption mainly mediated by methanogenesis and methanotrophic microorganisms, respectively (Oertel et al., 2016). Methanotrophic bacteria are taxonomically affiliated with the Proteobacteria and Verrucomicrobia phyla while methanogens belong to the Euryarchaeota phylum (Gagliano et al., 2014; Liu X. et al., 2017; Andressa, 2019). In the present study, there were higher relative abundances of Proteobacteria in RL and LL than that in SLL (Figure 4B) and significantly negative correlations between Proteobacteria and soil CH_4_ fluxes were also found (Supplementary Table S1). The higher Proteobacteria can lead to more CH_4_ oxidized in soil and thereby lead to lower CH_4_ fluxes in LL and RL (Figure 4B). Soil CH_4_ is produced under strictly anaerobic conditions. Although there was no information about Euryarchaeota in our study, the significantly higher soil water content (Figure 1) and bulk density (Xiao et al., 2020a) in SLL can lead to better anaerobic conditions. Thus, we can conclude that there will be more CH_4_ production in SLL than in LL and RL. This is another reason resulting in higher soil CH_4_ fluxes in SLL.

In addition to bacterial composition, soil temperature can also regulate the activity of methanotrophic microorganisms to control CH_4_ fluxes (Song et al., 2021). However, the effect of soil temperature on soil CH_4_ fluxes is also modified by soil water content (Oertel et al., 2016). Coinciding with previous studies (Chen et al., 2021; Wang et al., 2022), our study showed a significantly positive relationship between soil water content and soil CH_4_ fluxes (Supplementary Table S1). High soil water content in SLL can not only enhance anaerobic soil conditions which prefer soil CH_4_ production but also inhibit gas diffusion and ultimately lead to higher soil CH_4_ fluxes in SLL, compared with RL and LL. Meanwhile, soil temperature in LL was significantly higher than in RL, but the higher soil water content in LL led to similar soil CH_4_ fluxes between RL and LL. Moreover, soil NH_4_^+^ − N content is another vital factor affecting soil CH_4_ microbial processes in permafrost (Song et al., 2021). High NH_4_^+^ − N content can not only contribute to soil CH_4_ production by enhancing the abundance of methanogens but also can inhibit CH_4_ oxidation by competing for methane monooxygenase (Bédard and Knowles, 1989; Kuzyakov and Xu, 2013). Our study also documented the significant positive correlation between soil CH_4_ fluxes and soil NH_4_^+^ − N content, which is in line with previous studies (Song et al., 2021). Therefore, higher soil CH_4_ fluxes were found in SLL where there was higher soil NH_4_^+^ − N content. Further, it is worth noting that *Sphagnum* is a key species associated with methane oxidation (Kox et al., 2020). Thus, the *Sphagnum* in the SLL may lead to higher CH_4_ oxidation. However, the lowest soil CH_4_ oxidation in SLL may be due to the lower soil temperature and aboveground plant removal. The lower soil temperature can inhibit *Sphagnum* metabolism. It must also be remembered that the aboveground biomass of the vegetation was cut inside each collar. This treatment was necessary to avoid the effects of interference from vegetation on soil GHG fluxes, but it may have induced lower soil CH_4_ oxidation in SLL.

### Effect of understory species on soil N_2_O fluxes

N_2_O is produced mainly by incomplete nitrification and denitrification processes mediated by microbial metabolism (Hu et al., 2015; Oertel et al., 2016). Our study also showed that soil NH_4_^+^ − N and NO_3_^−^−N content, as the important substrates of nitrification and denitrification, both can significantly regulate soil N_2_O fluxes by affecting soil bacterial community (Figure 6C). Proteobacteria at the phylum level are recognized as an important factor in affecting soil N_2_O production from nitrification processes by regulating ammonium oxidation and nitrite accumulation (Yang et al., 2017). Ammonia oxidation is the rate-limiting step for the whole nitrification process, which is critical for N_2_O production from nitrification (Hu et al., 2015; Xue et al., 2021). Thus, the higher Proteobacteria can lead to higher soil N_2_O fluxes in RL and LL than that in SLL in the present study (Figure 4B). Meanwhile, nitrite accumulation can also induce higher N_2_O production by both nitrite reduction (NO_2_^−^ to NO) and further NO reduction (NO to N_2_O) in nitrifier denitrification pathways (Wrage et al., 2001; Supplementary Figure S2A), which is another reason resulting in higher N_2_O production in RL and LL.

Apart from nitrification, heterotrophic denitrification is also a vital pathway of soil N_2_O production which is conducted by denitrification microbes under oxygen-limited conditions (Oertel et al., 2016). However, given the soil net CH_4_ uptake and low soil NO_3_^−^−N content and nitrification rates (Xiao et al., 2022) among all types of larch forest, nitrite accumulation may be mainly from the ammonia oxidation pathway (Supplementary Figure S2A). This means that soil N_2_O production in our study may be mainly from nitrification-related pathways (ammonia oxidation and nitrifier denitrification). In both pathways, N_2_O production from ammonia oxidation usually occurs in high ammonia contents, low nitrite concentrations, and high nitrification rates (Wunderlin et al., 2012). In our study, there were high NH_4_^+^ − N contents, but the soil nitrification rates are low among the three types of larch forest (Xiao et al., 2022). Meanwhile, the higher abundance of ammonia-oxidizing bacteria (Nitrosomonadaceae) than nitrite oxidation bacteria (Nitrospire and Nitrobacter) also showed lower nitrification rates and higher nitrite accumulation (Supplementary Figure S2B). On the contrary, nitrifier denitrification can be a major pathway of N_2_O production under low pH conditions (Wrage et al., 2001). Thus, variations in soil N_2_O fluxes among three types of larch forest may be mainly from the nitrifier denitrification process in our study. Although there was the same pathway from NO_2_^−^ to N_2_O in both heterotrophic denitrification and nitrifier denitrification processes (Supplementary Figure S2B), the whole nitrifier denitrification processes are solely regulated by ammonia-oxidizing bacteria which may not need a strictly anaerobic process (Beaumont et al., 2004; Shaw et al., 2006). However, previous studies also speculate that denitrification is the dominant process of soil N_2_O production in our study area due to the high relationship between NO_3_^−^−N content and soil N_2_O fluxes (Gao et al., 2019; Wu et al., 2019). Thus, further studies are needed to confirm the soil N_2_O production mechanism from the gene level.

## Conclusion

Our study explored the vital role of understory species in regulating soil GHG fluxes among three types of larch forest. Shifts in understory species can alter soil GHG fluxes *via* their effects on soil environmental factors, soil properties, and soil bacterial community structure. Understory species regulated soil GHG fluxes mainly by affecting soil temperature and bacterial community among three types of larch forest. Meanwhile, soil NO_3_^−^−N, NH_4_^+^ − N content, and their interaction had significant indirect effects on soil CO_2_, CH_4_, and N_2_O fluxes *via* their effects on soil bacterial community, respectively. Our study also suggested that the variations in soil N_2_O fluxes were mainly from nitrifier denitrification during nitrification. These results further stressed the importance of understory species in boreal forests and explored the potential mechanisms of variations in soil GHG fluxes due to shifts in understory species in larch forests. In the context of global warming, given the unique characteristics of vegetation structure in boreal forests, the effect of understory on soil GHG fluxes should be given more attention, especially in forests that have the same tree layers.

## Data availability statement

The original contributions presented in the study are included in the article/Supplementary material, further inquiries can be directed to the corresponding authors.

## Author contributions

TC, XM, and BD designed the study. BD and RX conducted the study, processed the analysis, and wrote the original manuscript. BD, RX, ZG, and MG performed the field data collection and sample analysis. TC, XM, and MM reviewed and revised this manuscript. All authors contributed to the article and approved the submitted version.

## Funding

This study was financially supported by the National Key Research and Development Program of China (Grant No. 2021YFD2200405).

## Conflict of interest

The authors declare that the research was conducted in the absence of any commercial or financial relationships that could be construed as a potential conflict of interest.

## Publisher’s note

All claims expressed in this article are solely those of the authors and do not necessarily represent those of their affiliated organizations, or those of the publisher, the editors and the reviewers. Any product that may be evaluated in this article, or claim that may be made by its manufacturer, is not guaranteed or endorsed by the publisher.

## References

[ref1] Alves B. J. R. Smith K. A. Flores R. A. Cardoso A. S. Oliveira W. R. D. Jantalia C. P.. (2012). Selection of the most suitable sampling time for static chambers for the estimation of daily mean N_2_O flux from soils. Soil Biol. Biochem. 46, 129–135. doi: 10.1016/j.soilbio.2011.11.022

[ref2] Andressa M. V. (2019). Forest-to-Pasture Conversion in the Eastern Amazon: Impacts on the Soil Methane Microbial Communities. Piracicaba: University of São Paulo.

[ref3] Beaumont H. J. Lens S. I. Reijnders W. N. Westerhoff H. V. van Spanning R. J. (2004). Expression of nitrite reductase in Nitrosomonas europaea involves NsrR, a novel nitrite-sensitive transcription repressor. Mol. Microbiol. 54, 148–158. doi: 10.1111/j.1365-2958.2004.04248.x, PMID: 15458412

[ref4] Bédard C. Knowles R. (1989). Physiology, biochemistry, and specific inhibitors of CH_4_, NH_4_^+^, and CO oxidation by methanotrophs and nitrifiers. Microbiol. Rev. 53, 68–84. doi: 10.1128/mr.53.1.68-84.1989, PMID: 2496288 PMC372717

[ref5] Bond-Lamberty B. Gower S. T. Amiro B. Ewers B. E. (2011). Measurement and modelling of bryophyte evaporation in a boreal forest chronosequence. Ecohydrology 4, 26–35. doi: 10.1002/eco.118

[ref6] Chen Q. Long C. Chen J. Cheng X. (2021). Differential response of soil CO_2_, CH_4_, and N_2_O emissions to edaphic properties and microbial attributes following afforestation in Central China. Glob. Chang. Biol. 0, 1–13. doi: 10.1111/gcb.15826 34363712

[ref7] Chen X. Zhu H. Yan B. Shutes B. Xing D. Banuelos G.. (2020). Greenhouse gas emissions and wastewater treatment performance by three plant species in subsurface flow constructed wetland mesocosms. Chemosphere 239:124795. doi: 10.1016/j.chemosphere.2019.124795, PMID: 31520977

[ref8] De Long J. R. Dorrepaal E. Kardol P. Nilsson M.-C. Teuber L. M. Wardle D. A. (2016). Understory plant functional groups and litter species identity are stronger drivers of litter decomposition than warming along a boreal forest post-fire successional gradient. Soil Biol. Biochem. 98, 159–170. doi: 10.1016/j.soilbio.2016.04.009

[ref9] Doroski A. A. Helton A. M. Vadas T. M. (2019). Greenhouse gas fluxes from coastal wetlands at the intersection of urban pollution and saltwater intrusion: a soil core experiment. Soil Biol. Biochem. 131, 44–53. doi: 10.1016/j.soilbio.2018.12.023

[ref10] Duan B. Cai T. Man X. Xiao R. Gao M. Ge Z.. (2022). Different variations in soil CO_2_, CH_4_, and N_2_O fluxes and their responses to edaphic factors along a boreal secondary forest successional trajectory. Sci. Total Environ. 838:155983. doi: 10.1016/j.scitotenv.2022.155983, PMID: 35588825

[ref11] Duan B. Man X. Cai T. Xiao R. Ge Z. (2020). Increasing soil organic carbon and nitrogen stocks along with secondary forest succession in permafrost region of the Daxing'an mountains, Northeast China. Glob. Ecol. Conserv. 24:e01258. doi: 10.1016/j.gecco.2020.e01258

[ref12] Fierer N. Bradford M. A. Jackson R. B. (2007). Toward an ecological classification of soil bacteria. Ecology 88, 1354–1364. doi: 10.1890/05-1839 17601128

[ref13] Fu X. Yang F. Wang J. Di Y. Dai X. Zhang X.. (2015). Understory vegetation leads to changes in soil acidity and in microbial communities 27 years after reforestation. Sci. Total Environ. 502, 280–286. doi: 10.1016/j.scitotenv.2014.09.018, PMID: 25261818

[ref14] Gagliano A. L. D'Alessandro W. Tagliavia M. Parello F. Quatrini P. (2014). Methanotrophic activity and diversity of methanotrophs in volcanic geothermal soils at Pantelleria (Italy). Biogeosciences 11, 5865–5875. doi: 10.5194/bg-11-5865-2014

[ref15] Gao M. Man X. Duan B. (2021). Short-term effects of understory vegetation and litter on soil CO_2_ flux of natural forests in cold temperate zone of China. J. Beijing Forest. Univ. 43, 55–65. doi: 10.12171/j.1000-1522.20200249

[ref16] Gao D. Wang W. Gao W. Zeng Q. Liang H. (2022). Greenhouse gas fluxes response to autumn freeze-thaw period in continuous permafrost region of Daxing'an mountains, Northeast China. Environ. Sci. Pollut. Res. Int. 29, 63753–63767. doi: 10.1007/s11356-022-20371-2, PMID: 35461419

[ref17] Gao W. Yao Y. Liang H. Song L. Sheng H. Cai T.. (2019). Emissions of nitrous oxide from continuous permafrost region in the Daxing'an mountains, Northeast China. Atmos. Environ. 198, 34–45. doi: 10.1016/j.atmosenv.2018.10.045

[ref18] Habtewold J. Z. Helgason B. L. Yanni S. F. Janzen H. H. Ellert B. H. Gregorich E. G. (2020). Litter composition has stronger influence on the structure of soil fungal than bacterial communities. Eur. J. Soil Biol. 98:103190. doi: 10.1016/j.ejsobi.2020.103190

[ref19] Han M. Zhu B. (2020). Changes in soil greenhouse gas fluxes by land use change from primary forest. Glob. Chang. Biol. 26, 2656–2667. doi: 10.1111/gcb.14993, PMID: 31930624

[ref20] He C. Wang X. Wang D. Zhao Z. Wang F. Cheng L.. (2021). Impact of Spartina alterniflora invasion on soil bacterial community and associated greenhouse gas emission in the Jiuduansha wetland of China. Appl. Soil Ecol. 168:104168. doi: 10.1016/j.apsoil.2021.104168

[ref21] Hsieh S.-H. Yuan C.-S. Ie I.-R. Yang L. Lin H.-J. Hsueh M.-L. (2021). In-situ measurement of greenhouse gas emissions from a coastal estuarine wetland using a novel continuous monitoring technology: comparison of indigenous and exotic plant species. J. Environ. Manag. 281:111905. doi: 10.1016/j.jenvman.2020.111905, PMID: 33388713

[ref22] Hu H. W. Chen D. He J. Z. (2015). Microbial regulation of terrestrial nitrous oxide formation: understanding the biological pathways for prediction of emission rates. FEMS Microbiol. Rev. 39, 729–749. doi: 10.1093/femsre/fuv021, PMID: 25934121

[ref23] IPCC (2007). Climate change 2007: The physical science basis. Contribution of working group I to the fourth assessment report of the intergovernmental panel on climate change. Cambridge: Cambridge University Press.

[ref24] IPCC (2021). Climate change 2021: The physical science basis. Contribution of working group I to the sixth assessment report of the intergovernmental panel on climate change. Oxford, UK: Cambridge University Press.

[ref25] Jia W. Chen Y. Zhang J. Li C. Wang Q. Li G.. (2018). Response of greenhouse gas emissions and microbial community dynamics to temperature variation during partial nitrification. Bioresour. Technol. 261, 19–27. doi: 10.1016/j.biortech.2018.03.137, PMID: 29653330

[ref26] Johnstone J. F. Chapin F. S. Hollingsworth T. N. Mack M. C. Romanovsky V. Turetsky M. (2010). Fire, climate change, and forest resilience in interior Alaska. Can. J. For. Res. 40, 1302–1312. doi: 10.1139/X10-061

[ref27] Kolton M. Marks A. Wilson R. M. Chanton J. P. Kostka J. E. (2019). Impact of warming on greenhouse gas production and microbial diversity in anoxic peat from a sphagnum-dominated bog (Grand Rapids, Minnesota, United States). Front. Microbiol. 10:870. doi: 10.3389/fmicb.2019.00870, PMID: 31105668 PMC6498409

[ref28] Kox M. A. R. van den Elzen E. Lamers L. P. M. Jetten M. S. M. van Kessel M. (2020). Microbial nitrogen fixation and methane oxidation are strongly enhanced by light in sphagnum mosses. AMB Express 10:61. doi: 10.1186/s13568-020-00994-9, PMID: 32236738 PMC7109220

[ref29] Kuzyakov Y. Xu X. (2013). Competition between roots and microorganisms for nitrogen: mechanisms and ecological relevance. New Phytol. 198, 656–669. doi: 10.1111/nph.12235, PMID: 23521345

[ref30] Lee J. Zhou X. Seo Y. O. Lee S. T. Yun J. Yang Y.. (2022). Effects of vegetation shift from needleleaf to broadleaf species on forest soil CO_2_ emission. Sci. Total Environ. 856:158907. doi: 10.1016/j.scitotenv.2022.158907 36150592

[ref31] Li H.-f. (2010). Soil CH_4_ fluxes response to understory removal and N-fixing species addition in four forest plantations in southern China. J. For. Res. 21, 301–310. doi: 10.1007/s11676-010-0075-2

[ref32] Li X. Cheng S. Fang H. Yu G. Dang X. Xu M.. (2015). The contrasting effects of deposited NH_4_^+^ and NO_3_^−^ on soil CO_2_, CH_4_ and N_2_O fluxes in a subtropical plantation, southern China. Ecol. Eng. 85, 317–327. doi: 10.1016/j.ecoleng.2015.10.003

[ref33] Li H. Fu S. Zhao H. Xia H. (2010). Effects of understory removal and N-fixing species seeding on soil N_2_O fluxes in four forest plantations in southern China. Soil Sci. Plant Nutr. 56, 541–551. doi: 10.1111/j.1747-0765.2010.00498.x

[ref34] Li J. Zhu D. Wu Y. Xu N. Song J. (2018). Seasonal variation of emission fluxes of CO_2_, N_2_O and CH_4_ from four typical larch forests in Daxing’anling mountains of China. J. Centr. South Univ. Forest. Technol. 38, 95–102. doi: 10.14067/j.cnki.1673-923x.2018.11.014

[ref35] Lin Y. Cai T. Duan L. (2018). Snow hydrological characteristics of *Larix gmelinii* forest in northern Daxing’an mountains of northeastern China. J. Beijing Forest. Univ. 40, 72–80. doi: 10.13332/j.1000-1522.20170389

[ref36] Liu R. He Y. Zhou G. Shao J. Zhou L. Zhou H.. (2021). Mycorrhizal effects on decomposition and soil CO_2_ flux depend on changes in nitrogen availability during forest succession. J. Ecol. 109, 3929–3943. doi: 10.1111/1365-2745.13770

[ref37] Liu X. Lin T.-C. Yang Z. Vadeboncoeur M. A. Lin C. Xiong D.. (2017). Increased litter in subtropical forests boosts soil respiration in natural forests but not plantations of Castanopsis carlesii. Plant Soil 418, 141–151. doi: 10.1007/s11104-017-3281-2

[ref38] Liu Y. Shang Q. Wang L. Liu S. (2019). Effects of understory shrub biomass on variation of soil respiration in a temperate-subtropical transitional oak Forest. Forests 10:88. doi: 10.3390/f10020088

[ref39] Liu Y. Wang S. Li S. Deng Y. (2017). Advances in molecular ecology on microbial functional genes of carbon cycle. Microbiology China 44, 1676–1689. doi: 10.13344/j.microbiol.china.160941

[ref40] Liu X. P. Zhang W. J. Hu C. S. Tang X. G. (2014). Soil greenhouse gas fluxes from different tree species on Taihang Mountain, North China. Biogeosciences 11, 1649–1666. doi: 10.5194/bg-11-1649-2014

[ref41] Mazza G. Agnelli A. E. Lagomarsino A. (2021). The effect of tree species composition on soil C and N pools and greenhouse gas fluxes in a Mediterranean reforestation. J. Soil Sci. Plant Nutr. 21, 1339–1352. doi: 10.1007/s42729-021-00444-w

[ref42] Moqimzai O. (2020). Factors of global warming and its effects on the environment. Int. J. Res. Appl. Sci. Biotechnol. 7, 202–208. doi: 10.31033/ijrasb.7.6.30

[ref43] Muhammad I. Lv J. Z. Wang J. Ahmad S. Farooq S. Ali S.. (2022). Regulation of soil microbial community structure and biomass to mitigate soil greenhouse gas emission. Front. Microbiol. 13:868862. doi: 10.3389/fmicb.2022.868862, PMID: 35547151 PMC9083002

[ref44] Myers-Smith I. H. Forbes B. C. Wilmking M. Hallinger M. Lantz T. Blok D.. (2011). Shrub expansion in tundra ecosystems: dynamics, impacts and research priorities. Environ. Res. Lett. 6:045509. doi: 10.1088/1748-9326/6/4/045509

[ref45] Nilsson M.-C. Wardle D. A. (2005). Understory vegetation as a forest ecosystem driver: evidence from the northern Swedish boreal forest. Front. Ecol. Environ. 3, 421–428. doi: 10.1890/1540-9295(2005)003[0421:UVAAFE]2.0.CO;2

[ref46] Nordborg F. Nilsson U. Gemmel P. Örlander G. (2007). Carbon and nitrogen stocks in soil, trees and field vegetation in conifer plantations 10 years after deep soil cultivation and patch scarification. Scand. J. For. Res. 21, 356–363. doi: 10.1080/02827580600976615

[ref47] Oertel C. Matschullat J. Zurba K. Zimmermann F. Erasmi S. (2016). Greenhouse gas emissions from soils—a review. Geochemistry 76, 327–352. doi: 10.1016/j.chemer.2016.04.002

[ref48] Pan P. Zhao F. Ning J. Zhang L. Ouyang X. Zang H. (2018). Impact of understory vegetation on soil carbon and nitrogen dynamic in aerially seeded Pinus massoniana plantations. PLoS One 13:e0191952. doi: 10.1371/journal.pone.0191952, PMID: 29377926 PMC5788378

[ref001] Prévosto B. Helluy M. Gavinet J. Fernandez C. Balandier P. (2020). Microclimate in Mediterranean pine forests: What is the influence of the shrub layer?. Agric. For. Meteor. 282–283. doi: 10.1016/j.agrformet.2019.107856

[ref49] Quebbeman A. W. Menge D. N. L. Zimmerman J. Uriarte M. (2021). Topography and tree species improve estimates of spatial variation in soil greenhouse gas fluxes in a subtropical Forest. Ecosystems 25, 648–660. doi: 10.1007/s10021-021-00677-x

[ref50] Scharlemann J. P. W. Tanner E. V. J. Hiederer R. Kapos V. (2014). Global soil carbon: understanding and managing the largest terrestrial carbon pool. Carbon Manag. 5, 81–91. doi: 10.4155/cmt.13.77

[ref51] Shaw L. J. Nicol G. W. Smith Z. Fear J. Prosser J. I. Baggs E. M. (2006). Nitrosospira spp. can produce nitrous oxide via a nitrifier denitrification pathway. Environ. Microbiol. 8, 214–222. doi: 10.1111/j.1462-2920.2005.00882.x, PMID: 16423010

[ref52] Shih P. M. Ward L. M. Fischer W. W. (2017). Evolution of the 3-hydroxypropionate bicycle and recent transfer of anoxygenic photosynthesis into the Chloroflexi. Proc. Natl. Acad. Sci. U.S.A. 114, 10749–10754. doi: 10.1073/pnas.1710798114, PMID: 28923961 PMC5635909

[ref53] Song Y. Song C. Hou A. Sun L. Wang X. Ma X.. (2021). Temperature, soil moisture, and microbial controls on CO_2_ and CH_4_ emissions from a permafrost peatland. Environ. Prog. Sustain. Energy 40:e13693. doi: 10.1002/ep.13693

[ref54] Sponseller R. A. Gundale M. J. Futter M. Ring E. Nordin A. Nasholm T.. (2016). Nitrogen dynamics in managed boreal forests: recent advances and future research directions. Ambio 45, 175–187. doi: 10.1007/s13280-015-0755-4, PMID: 26744052 PMC4705067

[ref55] Trivedi P. Anderson I. C. Singh B. K. (2013). Microbial modulators of soil carbon storage: integrating genomic and metabolic knowledge for global prediction. Trends Microbiol. 21, 641–651. doi: 10.1016/j.tim.2013.09.005, PMID: 24139848

[ref56] Vance E. D. Brookes P. C. Jenkinson D. S. (1987). An extraction method for measuring soil microbial biomass C. Soil Biol. Biochem. 19, 703–707. doi: 10.1016/0038-0717(87)90052-6

[ref57] Villén-Peréz S. Heikkinen J. Salemaa M. Mäkipää R. (2020). Global warming will affect the maximum potential abundance of boreal plant species. Ecography 43, 801–811. doi: 10.1111/ecog.04720

[ref58] Wang X. Gao S. Chen J. Yao Z. Zhang X. (2022). Reducing soil CO_2_, CH_4_ and N_2_O emissions through management of harvest residues in Chinese fir plantation. For. Ecol. Manag. 511:120140. doi: 10.1016/j.foreco.2022.120140

[ref59] Wang S. Zuo X. Zhao X. Awada T. Luo Y. Li Y.. (2018). Dominant plant species shape soil bacterial community in semiarid sandy land of northern China. Ecol. Evol. 8, 1693–1704. doi: 10.1002/ece3.3746, PMID: 29435244 PMC5792618

[ref60] WMO (2019). WMO greenhouse gas bulletin-no.16: The state of greenhouse gases in the atmosphere based on global observations through 2019. 16.

[ref61] Wrage N. Velthof G. ML. VB Oenema O. (2001). Role of nitrifer denitrifcation in the production of nitrous oxide. Soil Biol. Biochem. 33, 1723–1732. doi: 10.1016/S0038-0717(01)00096-7

[ref62] Wu J. Xiong J. Hu C. Shi Y. Wang K. Zhang D. (2015). Temperature sensitivity of soil bacterial community along contrasting warming gradient. Appl. Soil Ecol. 94, 40–48. doi: 10.1016/j.apsoil.2015.04.018

[ref63] Wu X. Zang S. Ma D. Ren J. Chen Q. Dong X. (2019). Emissions of CO_2_, CH_4_, and N_2_O fluxes from forest soil in permafrost region of Daxing'an mountains, Northeast China. Int. J. Environ. Res. Public Health 16:2999. doi: 10.3390/ijerph16162999, PMID: 31434321 PMC6721090

[ref64] Wunderlin P. Mohn J. Joss A. Emmenegger L. Siegrist H. (2012). Mechanisms of N_2_O production in biological wastewater treatment under nitrifying and denitrifying conditions. Water Res. 46, 1027–1037. doi: 10.1016/j.watres.2011.11.080, PMID: 22227243

[ref65] Xiao H. Li Z. Dong Y. Chang X. Deng L. Huang J.. (2017). Changes in microbial communities and respiration following the revegetation of eroded soil. Agric. Ecosyst. Environ. 246, 30–37. doi: 10.1016/j.agee.2017.05.026

[ref66] Xiao R. Man X. Duan B. (2020a). Carbon and nitrogen stocks in three types of Larix gmelinii forests in Daxing’an mountains, Northeast China. Forests 11:305. doi: 10.3390/f11030305

[ref67] Xiao R. Man X. Duan B. Cai T. (2020b). Short-term litter manipulations have strong impact on soil nitrogen dynamics in Larix gmelinii forest of Northeast China. Forests 11:1205. doi: 10.3390/f11111205

[ref68] Xiao R. Man X. Duan B. Cai T. Ge Z. Li X.. (2022). Changes in soil bacterial communities and nitrogen mineralization with understory vegetation in boreal larch forests. Soil Biol. Biochem. 166:108572. doi: 10.1016/j.soilbio.2022.108572

[ref69] Xue S. Zhou L. Zhong M. Kumar Awasthi M. Mao H. (2021). Bacterial agents affected bacterial community structure to mitigate greenhouse gas emissions during sewage sludge composting. Bioresour. Technol. 337:125397. doi: 10.1016/j.biortech.2021.125397, PMID: 34139563

[ref70] Yang Y. Chen Z. Wang X. Zheng L. Gu X. (2017). Partial nitrification performance and mechanism of zeolite biological aerated filter for ammonium wastewater treatment. Bioresour. Technol. 241, 473–481. doi: 10.1016/j.biortech.2017.05.151, PMID: 28599226

[ref71] Yang J. Cooper D. J. Li Z. Song W. Zhang Y. Zhao B.. (2020). Differences in tree and shrub growth responses to climate change in a boreal forest in China. Dendrochronologia 63:125744. doi: 10.1016/j.dendro.2020.125744

[ref72] Yao Y. Shao M. Fu X. Wang X. Wei X. (2019). Effects of shrubs on soil nutrients and enzymatic activities over a 0–100 cm soil profile in the desert-loess transition zone. Catena 174, 362–370. doi: 10.1016/j.catena.2018.11.031

[ref73] Yigit N. Sevik H. Cetin M. Kaya N. (2016). “Determination of the effect of drought stress on the seed germination in some plant species,” in Water Stress in Plants. eds. Rahman I. Md. M.r. Begum Z. A. Hasegawa H. (IntechOpen), 43–62.

[ref74] Zhang J. Ji Y. Guo Y. Yin X. Li Y. Han J.. (2022). Responses of soil respiration and microbial community structure to fertilizer and irrigation regimes over 2 years in temperate vineyards in North China. Sci. Total Environ. 840:156469. doi: 10.1016/j.scitotenv.2022.156469, PMID: 35679935

[ref75] Zhang J. Li Y. Chang S. X. Jiang P. Zhou G. Liu J.. (2014). Understory vegetation management affected greenhouse gas emissions and labile organic carbon pools in an intensively managed Chinese chestnut plantation. Plant Soil 376, 363–375. doi: 10.1007/s11104-013-1996-2

[ref76] Zhang J. Li Y. Chang S. X. Qin H. Fu S. Jiang P. (2015). Understory management and fertilization affected soil greenhouse gas emissions and labile organic carbon pools in a Chinese chestnut plantation. For. Ecol. Manag. 337, 126–134. doi: 10.1016/j.foreco.2014.11.004

[ref002] Zhou X. Zhu H. Wen Y. Goodate U. M. Li X. You Y.. (2018). Effects of understory management on trade-offs and synergies between biomass carbon stock, plant diversity and timber production in eucalyptus plantations. For. Ecol. Manag. 410, 164–173. doi: 10.1016/j.foreco.2017.11.015

[ref77] Zhu Y. Merbold L. Leitner S. Xia L. Pelster D. E. Diaz-Pines E.. (2020). Influence of soil properties on N_2_O and CO_2_ emissions from excreta deposited on tropical pastures in Kenya. Soil Biol. Biochem. 140:107636. doi: 10.1016/j.soilbio.2019.107636

