# Supplementary material for: Understory species composition mediates soil greenhouse gas fluxes by affecting bacterial community diversity in boreal forests
Source: Front Microbiol. 2023 Jan 20;13:1090169. doi: 10.3389/fmicb.2022.1090169 (PMC9894877; doi:10.3389/fmicb.2022.1090169)
Supplement: Supplementary file 1 [file Data_Sheet_1.docx]

**TABLE S1丨**Correlation of soil GHG fluxes with selected soil factors measured among three types of larch forest. The bold number showed the significant correlations at *P* < 0.05 level.

| **Factors** | **CO_2_** | | **CH_4_** | | **N_2_O** | |  |
| --- | --- | --- | --- | --- | --- | --- | --- |
| ST | | **0.60** | | **-0.63** | | **0.77** | |
| SWC | | -0.39 | | **0.61** | | **-0.77** | |
| pH | | -0.15 | | -0.07 | | -0.09 | |
| NO3 | | -0.14 | | 0.43 | | **-0.74** | |
| NH4 | | -0.54 | | **0.77** | | **-0.85** | |
| MBC | | **0.96** | | **-0.93** | | 0.55 | |
| MBN | | **0.97** | | **-0.95** | | 0.58 | |
| Proteobacteria | | **0.74** | | **-0.66** | | **0.65** | |
| Actinobacteria | | 0.51 | | -0.57 | | 0.38 | |
| Acidobacteria | | -0.25 | | 0.29 | | -0.24 | |
| Chloroflexi | | **-0.79** | | **0.89** | | **-0.82** | |
| Verrucomicrobia | | 0.08 | | -0.36 | | 0.41 | |
| Gemmatimonadetes | | 0.38 | | -0.46 | | 0.50 | |
| Bacteroidetes | | **0.68** | | **-0.66** | | **0.75** | |


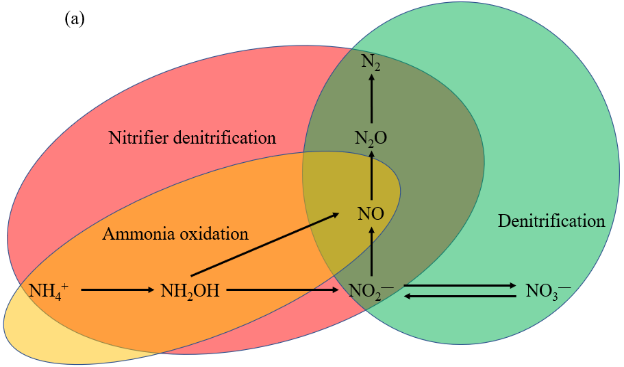


**FIGURE S1丨**The simplified schematic of pathways for soil N_2_O production (a) and comparison of the relative abundances of nitrifying bacteria among three types of larch forest (b). The yellow, red, and green indicated the N_2_O production from ammonia oxidation, nitrifier denitrification, and denitrification, respectively. Values are the mean ± SD. Different lowercase letters indicate statistically significant difference among different types of larch forest. Abbreviations: LL, Ledum palustre‐Larix gmelinii forest; RL, *Rhododendron dauricum*-*Larix gmelinii* forest; SLL, *Sphagnum*-*Bryum*-*Rhododendron tomentosum* forest.
